# Impact of renal function on the efficacy of low-voltage area ablation after pulmonary vein isolation: a sub-analysis of the SUPPRESS-AF trial

**DOI:** 10.1093/europace/euaf205

**Published:** 2025-09-02

**Authors:** Yasuhiro Matsuda, Masaharu Masuda, Toshiaki Mano, Takuya Tsujimura, Hiroyuki Uematsu, Hirotaka Ooka, Satoshi Kudo, Mizuki Ochi, Shin Okamoto, Takayuki Ishihara, Kiyonori Nanto, Yosuke Hata, Sho Nakao, Masaya Kusuda, Wataru Ariyasu, Akihiro Sunaga, Nobuaki Tanaka, Tetsuya Watanabe, Hitoshi Minamiguchi, Yasuyuki Egami, Takafumi Oka, Tomoko Minamisaka, Takashi Kanda, Masato Okada, Masato Kawasaki, Koji Tanaka, Nobuhiko Makino, Hirota Kida, Shungo Hikoso, Tomoharu Dohi, Koichi Inoue, Yohei Sotomi, Yasushi Sakata, Osaka Cardiovascular Conference (OCVC)-Arrhythmia Investigators, Yasuhiro Matsuda, Masaharu Masuda, Toshiaki Mano, Masato Okada, Nobuaki Tanaka, Koji Tanaka, Yuko Hirao, Masato Kawasaki, Tetsuya Watanabe, Takahisa Yamada, Takashi Kanda, Hitoshi Minamiguchi, Nobuhiko Makino, Yoshiharu Higuchi, Yasuharu Matsunaga, Yasuyuki Egami, Masami Nishino, Jun Tanouchi, Taiki Sato, Hirota Kida, Akihiro Sunaga, Tomoaki Nakano, Kentaro Ozu, Yohei Sotomi, Tomoharu Dohi, Katsuki Okada, Takafumi Oka, Toshihiro Takeda, Daisaku Nakatani, Shungo Hikoso, Yasushi Sakata, Miwa Miyoshi, Koichi Inoue, Yasushi Matsumura, Tomoko Minamisaka, Shiro Hoshida

**Affiliations:** Cardiovascular Center, Kansai Rosai Hospital, 3-1-69 Inabaso, Amagasaki 660-8511, Japan; Cardiovascular Center, Kansai Rosai Hospital, 3-1-69 Inabaso, Amagasaki 660-8511, Japan; Cardiovascular Center, Kansai Rosai Hospital, 3-1-69 Inabaso, Amagasaki 660-8511, Japan; Cardiovascular Center, Kansai Rosai Hospital, 3-1-69 Inabaso, Amagasaki 660-8511, Japan; Cardiovascular Center, Kansai Rosai Hospital, 3-1-69 Inabaso, Amagasaki 660-8511, Japan; Cardiovascular Center, Kansai Rosai Hospital, 3-1-69 Inabaso, Amagasaki 660-8511, Japan; Cardiovascular Center, Kansai Rosai Hospital, 3-1-69 Inabaso, Amagasaki 660-8511, Japan; Cardiovascular Center, Kansai Rosai Hospital, 3-1-69 Inabaso, Amagasaki 660-8511, Japan; Cardiovascular Center, Kansai Rosai Hospital, 3-1-69 Inabaso, Amagasaki 660-8511, Japan; Cardiovascular Center, Kansai Rosai Hospital, 3-1-69 Inabaso, Amagasaki 660-8511, Japan; Cardiovascular Center, Kansai Rosai Hospital, 3-1-69 Inabaso, Amagasaki 660-8511, Japan; Cardiovascular Center, Kansai Rosai Hospital, 3-1-69 Inabaso, Amagasaki 660-8511, Japan; Cardiovascular Center, Kansai Rosai Hospital, 3-1-69 Inabaso, Amagasaki 660-8511, Japan; Cardiovascular Center, Kansai Rosai Hospital, 3-1-69 Inabaso, Amagasaki 660-8511, Japan; Cardiovascular Center, Kansai Rosai Hospital, 3-1-69 Inabaso, Amagasaki 660-8511, Japan; Department of Cardiovascular Medicine, Osaka University Graduate School of Medicine, Suita, Japan; Cardiovascular Center, Sakurabashi Watanabe Advanced Healthcare Hospital, Osaka, Japan; Division of Cardiology, Osaka General Medical Center, Osaka, Japan; Department of Cardiovascular Medicine, Yao Municipal Hospital, Yao, Japan; Cardiovascular Division, Osaka Police Hospital, Osaka, Japan; Division of Cardiology, Osaka Rosai Hospital, Sakai, Japan; Department of Cardiovascular Medicine, Osaka University Graduate School of Medicine, Suita, Japan; Department of Cardiovascular Medicine, Yao Municipal Hospital, Yao, Japan; Cardiovascular Center, Kansai Rosai Hospital, 3-1-69 Inabaso, Amagasaki 660-8511, Japan; Cardiovascular Division, Osaka Police Hospital, Osaka, Japan; Cardiovascular Center, Sakurabashi Watanabe Advanced Healthcare Hospital, Osaka, Japan; Division of Cardiology, Osaka General Medical Center, Osaka, Japan; Cardiovascular Center, Sakurabashi Watanabe Advanced Healthcare Hospital, Osaka, Japan; Cardiovascular Division, Osaka Police Hospital, Osaka, Japan; Department of Cardiovascular Medicine, Osaka University Graduate School of Medicine, Suita, Japan; Department of Cardiovascular Medicine, Osaka University Graduate School of Medicine, Suita, Japan; Department of Cardiovascular Medicine, Nara Medical University, Kashihara, Japan; Department of Cardiovascular Medicine, Osaka University Graduate School of Medicine, Suita, Japan; Cardiovascular Division, National Hospital Organization Osaka National Hospital, Osaka, Japan; Department of Cardiovascular Medicine, Osaka University Graduate School of Medicine, Suita, Japan; Department of Cardiovascular Medicine, Osaka University Graduate School of Medicine, Suita, Japan

**Keywords:** Atrial fibrillation, Catheter ablation, Left atrial remodelling, Renal function, Chronic kidney disease

## Abstract

**Aims:**

The SUPPRESS-AF trial showed that pulmonary vein isolation (PVI) plus low-voltage area (LVA) ablation may reduce atrial fibrillation (AF) recurrence in some subgroups. Renal dysfunction is a cause of LVAs due to atrial cardiomyopathy and is also a risk factor for AF recurrence after catheter ablation. The aim of this study was to investigate the efficacy of LVA ablation after PVI stratified by renal function.

**Methods and results:**

This study was a sub-analysis of the SUPPRESS-AF trial, a multicentre, prospective, randomized, open-label trial. A total of 341 consecutive patients who underwent initial radiofrequency catheter ablation for persistent AF and whose LVAs were ≥5 cm^2^ were analysed. Patients were randomized to PVI alone (PVI-alone group) or LVA ablation after PVI [PVI + LVA-ablation (ABL) group]. Primary outcome was defined as the recurrence of atrial tachyarrhythmias during the 12 months following ablation. Estimated glomerular filtration rate (eGFR) was assessed before ablation, and patients were stratified by chronic kidney disease (CKD) stage. The mean eGFR was 60 ± 16 mL/min/1.73 m^2^, and 146 (43%) patients developed the primary outcome. In patients with CKD G1-2 (eGFR ≥ 60 mL/min/1.73 m^2^), freedom from the primary outcome was similar between the PVI + LVA-ABL and PVI-alone groups (53.1% vs. 55.3%, *P* = 0.59). In contrast, in patients with CKD G3a-5 (eGFR < 60 mL/min/1.73 m^2^), freedom from the primary outcome was significantly higher in the PVI + LVA-ABL group than in the PVI-alone group (69.1% vs. 43.3%; *P* = 0.004).

**Conclusion:**

In patients with renal dysfunction, LVA ablation after PVI reduced AF recurrence after radiofrequency catheter ablation for persistent AF.

## Graphical Abstract

**Graphical Abstract:**
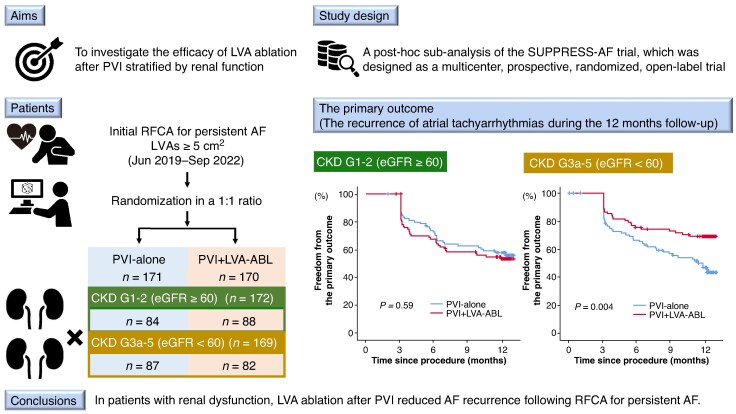
A total of 342 consecutive patients were prospectively enrolled in the SUPPRESS-AF study. Patients were initially randomized to pulmonary vein isolation (PVI) alone or PVI + low-voltage area (LVA)-ablation (ABL). One patient was excluded due to missing eGFR data. Finally, 171 patients in the PVI-alone group and 170 patients in the PVI + LVA-ABL group were analysed in this study. In patients with chronic kidney disease (CKD) G1-2, there was no significant difference in freedom from the primary outcome between the PVI-alone and PVI + LVA-ABL groups. Conversely, in patients with CKD G3a-5, freedom from the primary outcome was significantly higher in the PVI + LVA-ABL group than in the PVI-alone group. LVAs, low-voltage areas; PVI, pulmonary vein isolation; RFCA, radiofrequency catheter ablation; AF, atrial fibrillation; ABL, ablation; CKD, chronic kidney disease; eGFR, estimated glomerular filtration rate.

What’s new?

- This sub-analysis of the SUPPRESS-AF trial investigated the efficacy of low-voltage area (LVA) ablation after pulmonary vein isolation (PVI) stratified by renal function.
- In radiofrequency catheter ablation for persistent atrial fibrillation (AF), LVA ablation after PVI did not reduce AF recurrence in patients without renal dysfunction (estimated glomerular filtration rate < 60 mL/min/1.73 m^2^). In contrast, LVA ablation after PVI reduced AF recurrence in patients with renal dysfunction.
- These findings suggest that while routine LVA ablation after PVI may not be recommended in patients without renal dysfunction, aggressive substrate modification by LVA ablation may be considered in those with renal dysfunction.

## Introduction

Catheter ablation is an effective and rapidly advancing therapy for atrial fibrillation (AF).^1^ Although pulmonary vein isolation (PVI) is commonly performed as standard therapy of AF ablation,^1^ many patients experience AF recurrence after catheter ablation by PVI alone, especially those with persistent AF.^1^ Although additional ablation other than PVI is also performed to modify the AF substrate,^2,3^ no strategy for left atrial additional ablation other than PVI has yet been established.^1^

Left atrial low-voltage areas (LVAs), which reflect atrial remodelling caused by atrial cardiomyopathy,^4^ are also associated with poor rhythm outcomes after catheter ablation for AF.^5,6^ Low-voltage area ablation was developed as a patient-tailored ablation that ablated LVAs, either by encircling or cluster ablation, with the aim of eliminating LVAs.^1,7,8^ Although the SUPPRESS-AF trial showed that AF recurrence in patients undergoing persistent AF ablation was not statistically reduced by the routine use of LVA ablation in addition to PVI,^9^ there was nevertheless a greater reduction in AF recurrence in the PVI plus LVA ablation group than in the PVI-alone group in some subgroups, such as those with older age, a large left atrial diameter, and a large size of LVAs.^9^

Chronic kidney disease (CKD) is a risk factor for many cardiovascular diseases.^10^ Chronic kidney disease induces atrial remodelling via a number of factors, including electrolyte imbalance, increased sympathetic activity, and inflammation.^11,12,13^ A previous study showed that the severity of CKD is correlated with the prevalence of LVAs.^14^ However, the association of renal function with the efficacy of LVA ablation after PVI has not been clarified.

We have hypothesized that LVA ablation after PVI would be effective in patients with renal dysfunction. Here, we evaluated the efficacy of LVA ablation after PVI stratified by renal function.

## Methods

### Patients and assessment of renal function

This study was conducted as a *post hoc* sub-analysis of the Efficacy and Safety of Left Atrial Low-voltage Area Guided Ablation for Recurrence Prevention Compared to Pulmonary Vein Isolation Alone in Patients with Persistent Atrial Fibrillation trial (SUPPRESS-AF trial), which was designed as a multicentre, prospective, randomized, open-label trial.^9,15^ This sub-analysis included 342 consecutive patients with persistent AF who underwent indexed radiofrequency catheter ablation. Persistent AF was defined as AF with a sustained episode lasting ≥7 days at enrolment^15^ and long-standing persistent AF as AF with a continuous AF episode lasting more than 1 year.^1^

Patients first underwent PVI during the procedure, followed by voltage mapping. If the size of LVAs detected by voltage mapping was ≥5 cm^2^, patients were randomized 1:1 by an online system to a strategy of PVI alone (PVI-alone group) or LVA ablation after PVI [PVI + LVA-ablation (ABL) group].^9,15^ Exclusion criteria were as follows: dialysis, left atrial diameter ≥ 55 mm, history of cardiac surgery, valvular AF, age < 20 years, pregnancy, history of thromboembolism (stroke, transient ischaemic attack, or systemic embolism) within the previous 6 months, and physician assessment as ineligible for the SUPPRESS-AF trial.^9,15^ In this sub-analysis, patients with missing estimated glomerular filtration rate (eGFR) data were also excluded.

In this study, patients were also stratified by renal function. Renal function was assessed most recently within 90 days prior to catheter ablation using creatinine-based eGFR.^10^ Based on the Kidney Disease Improving Global Outcomes (KDIGO) 2024 guideline,^10^ eGFR was rounded to the nearest whole number, and eGFR categories were defined as G1 (normal or high), ≥90 mL/min/1.73 m^2^; G2 (mildly decreased), 60–89 mL/min/1.73 m^2^; G3a (mildly to moderately decreased), 45–59 mL/min/1.73 m^2^; G3b (moderately to severely decreased), 30–44 mL/min/1.73 m^2^; G4 (severely decreased), 15–29 mL/min/1.73 m^2^; and G5 (renal failure), <15 mL/min/1.73 m^2^. Patients were also divided into two groups by CKD stage, namely CKD G1-2 (eGFR ≥ 60 mL/min/1.73 m^2^) and CKD G3a-5 (eGFR < 60 mL/min/1.73 m^2^), on the basis that eGFR < 60 mL/min/1.73 m^2^ is a criterion for CKD.^10^

This study was funded through IIS grant by Biosense Webster, Inc., part of Johnson & Johnson MedTech. Both the SUPPRESS-AF trial and this study adhered to the Declaration of Helsinki, and written informed consent for catheter ablation and enrolment in the SUPPRESS-AF trial was obtained from all patients. The SUPPRESS-AF trial was registered with UMIN-CTR (UMIN000035940), and the protocol was approved by the institutional review board at each participating hospital.

### Catheter ablation procedure

As described in previous publications of the SUPPRESS-AF trial,^9,15^ all procedures were performed with a single 3-D mapping system (CARTO3; Biosense Webster, Inc., Diamond Bar CA, USA). Initially, ipsilateral circumferential PVI was performed using an open-irrigated linear ablation catheter with a 3.5 mm tip (Thermocool SmartTouch; Biosense Webster, Inc.). Ablation lesion quality during PVI was assessed using the ablation index (AI).^16^ If AF persisted after PVI, electrical cardioversion was performed before voltage mapping.

After completion of PVI, voltage mapping was performed in the left atrium with pacing from the right atrium. A multi-electrode mapping catheter (Pentaray; Biosense Webster, Inc.) or 20-pole circular catheter (Lasso Nav; Biosense Webster, Inc.) was used for voltage mapping. Low-voltage areas were defined as areas with a bipolar peak-to-peak voltage < 0.5 mV, although areas with a bipolar peak-to-peak voltage < 0.05 mV were considered as scar lesions. Low-voltage area size was measured manually in each case, and patients with a total LVA size ≥ 5 cm^2^ were randomized to either the PVI + LVA-ABL group or PVI-alone group. In contrast, patients with a total LVA area < 5 cm^2^ were excluded.

Cluster ablation of all LVAs was performed in the PVI + LVA-ABL group. However, LVAs in the left atrial posterior wall could be isolated by roof line linear ablation and floor line linear ablation to avoid oesophageal injury. The quality of the ablation lesion in LVA ablation was also assessed using AI.^16^ Omission of LVA ablation was allowed if LVAs were located at a region where ablation was dangerous, such as the atrial septum near Bachmann’s bundle or the His bundle and posterior wall near the oesophagus.

After LVA ablation, tachyarrhythmia inducibility was assessed by atrial burst pacing and isoproterenol infusion. If atrial tachycardia/flutter and non-PV foci were detected, these could be ablated at the discretion of the operator.

### Patient follow-up

Atrial fibrillation recurrence after ablation was followed for 12 months. Routine follow-up was performed at 3, 6, 9, and 12 months after catheter ablation. Twelve-lead electrocardiography was performed at each follow-up, and Holter electrocardiography was performed at the 6- and 12-month follow-ups. Further, 30 s rhythm checks were performed with a portable ECG (HCG 901 or HCG 801; Omron, Kyoto, Japan) twice every day between 6 and 12 months, and symptom-driven rhythm checks were done with a portable ECG at the discretion of the physician.

Primary endpoint was defined as the recurrence of atrial tachyarrhythmia without antiarrhythmic agents during the 12 months following the initial catheter ablation procedure. The post-ablation blanking period was set at 3 months. Atrial tachyarrhythmia recurrence was defined as any AF or atrial tachycardia detected by electrocardiogram or AF or atrial tachycardia lasting ≥30 s detected by Holter ECG monitoring ≥3 months after the catheter ablation procedure.

Repeat ablation was performed for recurrent atrial tachyarrhythmia at the discretion of the attending physician. During repeat ablation procedures, LVA ablation was not allowed in the PVI-alone group. Conversely, repeat LVA ablation could be performed in the PVI + LVA-ABL group.

Secondary endpoints were the recurrence of atrial tachyarrhythmias after the last ablation procedure and a composite of all-cause death, symptomatic stroke, and bleeding. Each event was assessed separately during the 12-month follow-up period. Procedure-related adverse events were also assessed for 6 months after the index catheter ablation procedure. Bleeding was defined as hospitalization for bleeding and/or major bleeding according to the ISTH bleeding criteria.^17^

### Statistical analysis

In this study, continuous data are reported as mean ± standard deviation or median (1st-3rd quartiles) and categorical data as absolute values (percentages). Significance tests were performed using the unpaired Student’s *t*-test, Mann–Whitney *U* test, or Kruskal–Wallis test for continuous variables and χ^2^ test or Fisher’s exact test for categorical variables. Kaplan–Meier analysis and the log-rank test were used to assess the association between outcomes and renal function. For patients with CKD G1-2 and those with CKD G3a-5, subgroup analysis of the primary endpoint was also performed using Cox proportional hazards regression analysis. Subgroup types were determined based on the subgroup with a significantly greater reduction in the primary endpoint in the PVI + LVA-ABL group in the main publication of the SUPPRESS-AF trial.^9^ All statistical analyses in this study were performed using commercial software (SPSS™; SPSS, Inc., Chicago IL, USA).

## Results

### Patient flow chart and patient characteristics

**Figure 1:**
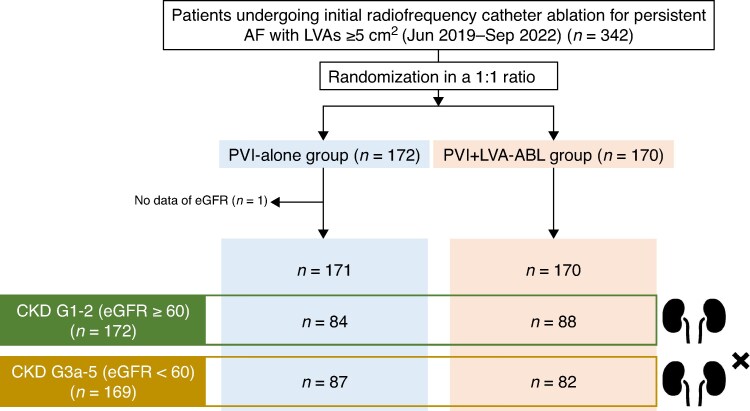
Patient flow chart. A total of 342 consecutive patients were prospectively enrolled in the SUPPRESS-AF study. Patients were initially randomized to PVI alone or PVI + LVA-ABL. One patient was excluded due to missing eGFR data. Finally, 171 patients in the PVI-alone group and 170 patients in the PVI + LVA-ABL group were analysed. AF, atrial fibrillation; LVAs, low-voltage areas; PVI, pulmonary vein isolation; ABL, ablation; eGFR, estimated glomerular filtration rate.

*Figure 1* shows the patient flow chart of this study. From eight institutions, 342 consecutive patients with ≥5 cm^2^ LVAs were prospectively enrolled between June 2019 and September 2022. Patients were randomized 1:1 to the PVI-alone or PVI + LVA-ABL group. After excluding one patient with missing eGFR data, 171 patients in the PVI-alone group and 170 patients in the PVI + LVA-ABL group were followed for 12 months.

Patient characteristics are shown in *Table 1*. Chronic kidney stage was G1 in 14 (4%) patients, G2 in 158 (46%), G3a in 122 (36%), G3b in 39 (11%), G4 in 7 (2%), and G5 in 1 (0.3%). One hundred and seventy-two (50%) patients had CKD G1-2 and 169 (50%) had CKD G3a-5. Patients with CKD G3a-5 were older and had a higher CHA_2_DS_2_-VA score as well as higher creatinine and higher N-terminal pro-brain natriuretic peptide levels than patients with CKD G1-2. Additionally, patients with CKD G3a-5 had a higher prevalence of congestive heart failure and were more likely to use beta antagonists and diuretics than those with CKD G1-2. Conversely, patients with CKD G3a-5 had a lower eGFR than those with CKD G1-2.

**Table 1:** Patient characteristics

Variable	All (*n* = 341)	CKD G1-2 (*n* = 172)	CKD G3a-5 (*n* = 169)	*P*
Age, years old	74 ± 6	73 ± 7	76 ± 6	<0.001
Female, *n* (%)	167 (49)	86 (50)	81 (48)	0.70
Long-standing persistent AF, *n* (%)	70 (21)	35 (20)	35 (21)	0.93
Duration of AF, *n* (%)	4 (2–10)	4 (2–11)	4 (2–10)	0.84
Body mass index, kg/m^2^	24 ± 4	23 ± 4	24 ± 4	0.26
CHA_2_DS_2_-VA score	3.0 ± 1.3	2.7 ± 1.4	3.3 ± 1.2	<0.001
Congestive heart failure, *n* (%)	104 (31)	42 (24)	62 (37)	0.01
NYHA class	1 (1–1)	1 (1–1)	1 (1–2)	0.06
Hypertension, *n* (%)	242 (71)	117 (68)	125 (74)	0.23
Diabetes mellitus, *n* (%)	77 (23)	37 (22)	40 (24)	0.63
Thromboembolism, *n* (%)	16 (5)	8 (5)	8 (5)	0.97
COPD, *n* (%)	17 (5)	6 (4)	11 (7)	0.20
Thyroid disease, *n* (%)	17 (5)	7 (4)	10 (6)	0.43
Direct oral anticoagulant, *n* (%)	328 (96)	166 (97)	162 (96)	0.75
Antiarrhythmic agent, *n* (%)	22 (7)	11 (6)	11 (7)	0.97
Renin–angiotensin system inhibitor, *n* (%)	135 (40)	61 (36)	74 (44)	0.12
Beta-blocker, *n* (%)	178 (52)	75 (44)	103 (61)	0.001
Calcium channel blocker, *n* (%)	165 (48)	83 (48)	82 (49)	0.96
Diuretics, *n* (%)	115 (34)	43 (25)	72 (43)	0.001
Haemoglobin, g/L	13.6 ± 1.5	13.6 ± 1.3	13.5 ± 1.7	0.34
NT-ProBNP, ng/L	1092 (681–1737)	913 (566–1261)	1460 (897–2087)	<0.001
Creatinine, mg/dL	0.9 ± 0.3	0.7 ± 0.1	1.1 ± 0.3	<0.001
eGFR, mL/min/1.73 m^2^	60 ± 16	73 ± 11	48 ± 9	<0.001
Left ventricular ejection fraction, %	57 ± 11	57 ± 10	56 ± 11	0.17
Left atrial diameter, mm	44 ± 5	44 ± 6	44 ± 5	0.66

CKD, chronic kidney disease; AF, atrial fibrillation; NYHA, New York Heart Association; COPD, chronic obstructive pulmonary disease; NT-proBNP, N-terminal pro-brain natriuretic peptide; eGFR, estimated glomerular filtration rate.

### Procedural characteristics and size of low-voltage areas

The procedural characteristics are shown in *Table 2*. Patients with CKD G3a-5 had a larger LVA size than those with CKD G1-2 [16 (10–25) cm^2^ vs. 12 (8–21) cm^2^; *P* = 0.008]. In addition, LVA size increased with increasing CKD severity (CKD G1, 9 (6–20) cm^2^; CKD G2, 12 (8–22) cm^2^; CKD G3a, 15 (10–25) cm^2^; CKD G3b, 16 (10–22) cm^2^; CKD G4–5, 29 (12–53) cm^2^; *P* = 0.03]. In patients with CKD G1-2, procedure time was longer in the PVI + LVA-ABL group than in the PVI-alone group. In patients with CKD G3a-5, atrial tachycardia ablations other than CTI were performed more frequently in the PVI + LVA-ABL group than in the PVI-alone group.

**Table 2:** Procedural characteristics

Variable	All (*n* = 341)	CKD G1-2 (*n* = 172)		*P*	CKD G3a-5 (*n* = 169)		*P*
		PVI-alone (*n* = 84)	PVI + LVA-ABL (*n* = 88)		PVI-alone (*n* = 87)	PVI + LVA-ABL (*n* = 82)	
Procedural time, min	178 ± 68	156 ± 59	192 ± 73	0.001	172 ± 59	192 ± 73	0.052
Deflectable sheath, *n* (%)	268 (79)	67 (80)	68 (77)	0.69	67 (77)	66 (81)	0.58
First-pass isolation, *n* (%)							
All, *n* (%)	275 (81)	72 (86)	72 (82)	0.49	65 (75)	66 (81)	0.37
Left PV, *n* (%)	300 (88)	76 (91)	79 (90)	0.88	73 (84)	72 (88)	0.47
Right PV, *n* (%)	298 (87)	77 (92)	77 (88)	0.37	74 (85)	70 (85)	0.96
Total size of LVAs, cm^2^	13 (9–24)	12 (8–24)	12 (8–18)	0.53	15 (9–25)	16 (10–26)	0.73
Total surface area of left atrium, cm^2^	161 (134–191)	162 (132–186)	163 (139–195)	0.40	154 (134–184)	168 (135–199)	0.29
Total no. of mapping points of left atrium, points	1678 (1274–2301)	1619 (1201–2137)	1632 (1254–2208)	0.54	1808 (1393–2319)	1749 (1389–2386)	0.91
LVA ablation, *n* (%)	170 (50)	0 (0)	88 (100)	<0.001	0 (0)	82 (100)	<0.001
Completion of LVA ablation, *n* (%)	133 (78)	–	75 (85)	–	–	58 (71)	–
CTI linear ablation, *n* (%)	84 (25)	18 (21)	18 (21)	0.88	23 (26)	25 (31)	0.56
AT ablation other than CTI, *n* (%)	40 (12)	9 (11)	15 (17)	0.23	3 (3)	13 (16)	0.008
Superior vena cava ablation, *n* (%)	8 (2)	1 (1)	2 (2)	1.00	4 (5)	1 (1)	0.37
Non-PV trigger ablation, *n* (%)	18 (5)	6 (7)	3 (3)	0.32	6 (7)	3 (4)	0.50

CKD, chronic kidney disease; PVI, pulmonary vein isolation; LVA, low-voltage area; PV, pulmonary vein; CTI, cavotricuspid isthmus; AT, atrial tachycardia.

### Atrial fibrillation recurrence and renal function

**Figure 2:**
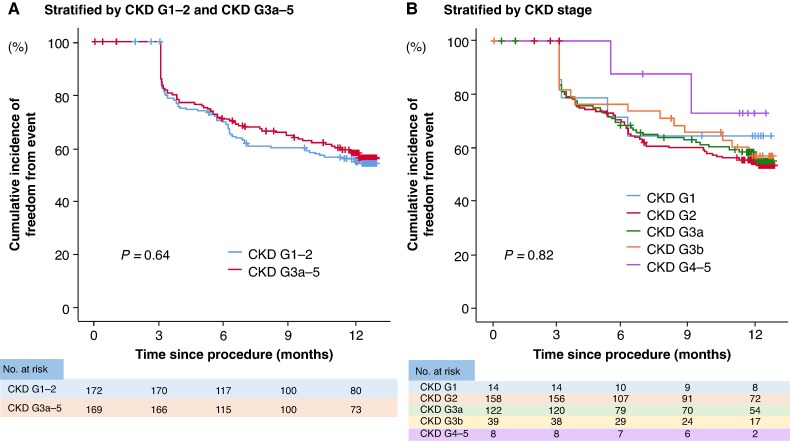
Association between the primary outcome and renal function. (*A*) There was no difference in freedom from the primary outcome between patients with CKD G1-2 and those with CKD G3a-5. (*B*) There was no difference in freedom from the primary outcome between patients by stage of CKD. CKD, chronic kidney disease.

A total of 146 patients (43%) experienced the primary outcome during the 12-month follow-up period. There was no difference in the incidence of freedom from the primary outcome between patients with CKD G1-2 and CKD G3a-5 (54.2% vs. 56.0%; *P* = 0.64) (*Figure 2A*). There was also no difference in the incidence of freedom from the primary outcome between patients by CKD stage (CKD G1, 64.3%; CKD G2, 53.3%; CKD G3a, 54.9%; CKD G3b, 56.7%; CKD G4-5, 72.9%; *P* = 0.82) (*Figure 2B*).

**Figure 3:**
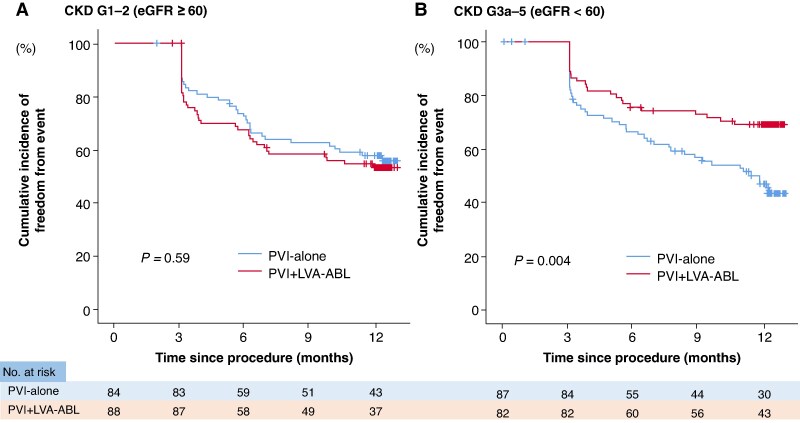
Association between the primary outcome and ablation strategy by CKD G1-2 vs. CKD G3a-5. (*A*) In patients with CKD G1-2, there was no significant difference in freedom from the primary outcome between the PVI-alone and PVI + LVA-ABL groups. (*B*) In patients with CKD G3a-5, freedom from the primary outcome was significantly higher in the PVI + LVA-ABL group than in the PVI-alone group. eGFR, estimated glomerular filtration rate; CKD, chronic kidney disease; PVI, pulmonary vein isolation; LVA, low-voltage area; ABL, ablation.

In patients with CKD G1-2, freedom from the primary outcome was similar between the PVI + LVA-ABL and PVI-alone groups (53.1% vs. 55.3%; *P* = 0.59) (*Figure 3A*). In contrast, in patients with CKD G3a-5, freedom from the primary outcome was significantly higher in the PVI + LVA-ABL group than in the PVI-alone group (69.1% vs. 43.3%; *P* = 0.004) (*Figure 3B*). The interaction between CKD G1-2 or CKD G3a-5 and ablation strategy on the primary outcome was significant (*P* = 0.02). Comparison of freedom from the primary outcome among groups stratified by CKD stage is also shown in Supplementary material online, *Figure S1*.

Similarly, in patients with CKD G1-2, there was no difference in freedom from AF recurrence after the last ablation between the PVI-alone and PVI + LVA-ABL groups (61.7% vs. 60.3%, *P* = 0.71) (see Supplementary material online, *Figure S2A*). In contrast, in patients with CKD G3a-5, freedom from AF recurrence after the last ablation was significantly higher in the PVI + LVA-ABL than in the PVI-alone group (76.1% vs. 51.8%; *P* = 0.007) (see Supplementary material online, *Figure S2B*).

### Subgroup analysis of the primary outcome

**Figure 4:**
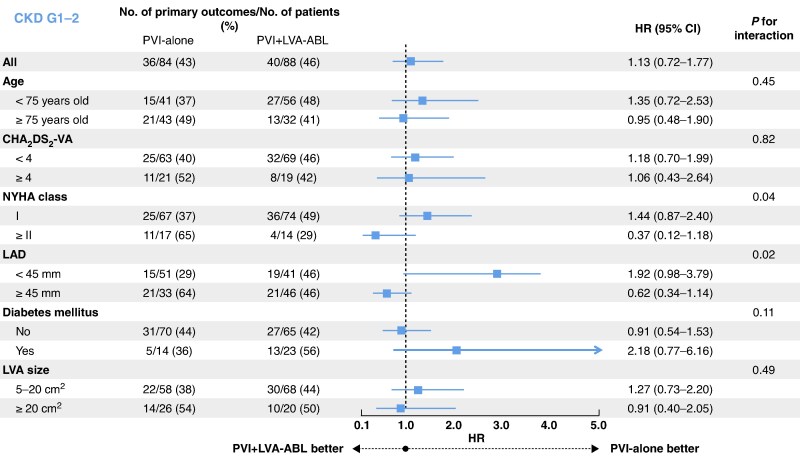
Subgroup analysis of the primary endpoint in patients with CKD G1-2 (eGFR ≥ 60 mL/min/1.73 m^2^). In patients with CKD G1-2, no subgroup in the PVI + LVA-ABL group showed a reduction in primary endpoint compared to the PVI-alone group. CKD, chronic kidney disease; PVI, pulmonary vein isolation; LVA, low-voltage area; ABL, ablation; NYHA, New York Heart Association; LAD, left atrial diameter; eGFR, estimated glomerular filtration rate.

**Figure 5:**
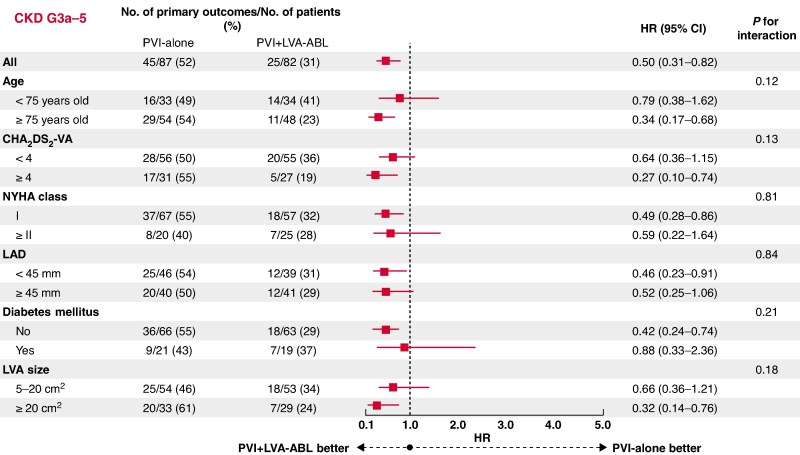
Subgroup analysis of the primary endpoint in patients with CKD G3a-5 (eGFR < 60 mL/min/1.73 m^2^). In patients with CKD G3a-5, prevention of the primary endpoint was greater in the PVI + LVA-ABL group compared to the PVI-alone group in patients aged ≥75 years and those with CHA_2_DS_2_-VA score ≥ 4, NYHA Class I, LAD < 45 mm, without diabetes mellitus, and with LVA ≥ 20 cm^2^. CKD, chronic kidney disease; PVI, pulmonary vein isolation; LVA, low-voltage area; ABL, ablation; NYHA, New York Heart Association; LAD, left atrial diameter; eGFR, estimated glomerular filtration rate.

A subgroup analysis of the primary endpoint was performed in patients with CKD G1-2 and those with CKD G3a-5. In patients with CKD G1-2, there was no subgroup with a reduction in the primary endpoint in the PVI + LVA-ABL group compared to the PVI-along group (*Figure 4*). By contrast, in patients with CKD G3a-5, an advantage in prevention of the primary endpoint in the PVI + LVA-ABL group compared to the PVI-alone group was observed in patients aged ≥75 years old, as well as those with any of a CHA_2_DS_2_-VA score ≥ 4, New York Heart Association (NYHA) Class I, left atrial diameter < 45 mm, without diabetes mellitus, and LVA ≥ 20 cm^2^ (*Figure 5*).

### Safety outcomes

The incidence of the composite endpoint of all-cause death, symptomatic stroke, and bleeding was similar between patients with CKD G1-2 and those with CKD G3a-5 [10 (6%) vs. 6 (4%); *P* = 0.32]. Individually assessed all-cause death [3 (2%) vs. 1 (1%); *P* = 0.62], symptomatic stroke [6 (4%) vs. 1 (1%); *P* = 0.12], and bleeding [4 (2%) vs. 4 (2%); *P* = 1.00] were also similar between patients with CKD G1-2 and those with CKD G3a-5.

Even when stratified by CKD G1-2 and CKD G3a-5, there was no difference in the composite endpoint, or in any of its components when assessed individually (i.e. all-cause death, symptomatic stroke, and bleeding), between the PVI-alone and PVI + LVA-ABL groups (see Supplementary material online, *Table S1*).

The rate of procedure-related complications was similar between patients with CKD G1-2 and CKD G3a-5 [7 (4%) vs. 8 (5%); *P* = 0.80]. Procedure-related complications stratified by CKD G1-2 and CKD G3a-5 are shown in *Table 3*. In both patients with CKD G1-2 and CKD G3a-5, there was no statistically significant difference in the overall procedure-related complication rate or rates of individual complications between the PVI-alone group and the PVI + LVA-ABL group.

**Table 3:** Procedure-related complications

Variable	All (*n* = 341)	CKD G1-2 (*n* = 172)		*P*	CKD G3a-5 (*n* = 169)		*P*
		PVI-alone (*n* = 84)	PVI + LVA-ABL (*n* = 88)		PVI-alone (*n* = 87)	PVI + LVA-ABL (*n* = 82)	
All, *n* (%)	15 (4)	2 (2)	5 (6)	0.44	2 (2)	6 (7)	0.16
Haematoma, *n* (%)	2 (0.6)	0 (0)	2 (2)	0.50	0 (0)	0 (0)	–
Haemorrhage,^a^ *n* (%)	0 (0)	0 (0)	0 (0)	–	0 (0)	0 (0)	–
Systemic thromboembolism, *n* (%)	1 (0.3)	0 (0)	0 (0)	–	1 (1)	0 (0)	1.00
Pneumothorax, *n* (%)	0 (0)	0 (0)	0 (0)	–	0 (0)	0 (0)	–
Atrial venous fistula, *n* (%)	0 (0)	0 (0)	0 (0)	–	0 (0)	0 (0)	–
Pericarditis, *n* (%)	1 (0.3)	1 (1)	0 (0)	0.49	0 (0)	0 (0)	–
Cardiac tamponade, *n* (%)	1 (0.3)	1 (1)	0 (0)	0.49	0 (0)	0 (0)	–
Phrenic nerve paralysis, *n* (%)	0 (0)	0 (0)	0 (0)	–	0 (0)	0 (0)	–
Atrioventricular block, *n* (%)	0 (0)	0 (0)	0 (0)	–	0 (0)	0 (0)	–
Sick sinus syndrome, *n* (%)	0 (0)	0 (0)	0 (0)	–	0 (0)	0 (0)	–
Pulmonary hypertension with pulmonary vein stenosis, *n* (%)	0 (0)	0 (0)	0 (0)	–	0 (0)	0 (0)	–
Atrio-oesophageal fistula, *n* (%)	1 (0.3)	0 (0)	1 (1)	1.00	0 (0)	0 (0)	–
Acute gastric dilatation, *n* (%)	0 (0)	0 (0)	0 (0)	–	0 (0)	0 (0)	–
Infection, *n* (%)	0 (0)	0 (0)	0 (0)	–	0 (0)	0 (0)	–
Heart failure, *n* (%)	6 (2)	1 (1)	1 (1)	1.00	0 (0)	4 (5)	0.053
Allergy, *n* (%)	0 (0)	0 (0)	0 (0)	–	0 (0)	0 (0)	–
Others, *n* (%)	4 (1)	0 (0)	1 (1)	1.00	1 (1)	2 (2)	0.61

CKD, chronic kidney disease; PVI, pulmonary vein isolation; LVA, low-voltage area.

^a^Haemorrhage consisted of gastrointestinal bleeding and intracranial haemorrhage.

## Discussion

This study, a sub-analysis of the multicentre, prospective, randomized, open-label SUPPRESS-AF trial, was conducted in 341 patients who underwent initial radiofrequency catheter ablation for persistent AF. Our aim was to evaluate the efficacy of LVA ablation after PVI in patients stratified by renal function. During 12 months of follow-up, we found no significant difference in freedom from AF recurrence between the PVI + LVA-ABL group and PVI-alone group in patients with CKD G1-2. In contrast, freedom from AF recurrence was significantly higher in the PVI + LVA-ABL group than in the PVI-alone group in patients with CKD G3a-5. To our knowledge, this is the first clinical study to evaluate the efficacy of LVA ablation after PVI in patients stratified by renal function.

### Association between the efficacy of low-voltage area ablation and renal dysfunction

Low-voltage areas are clinical surrogate markers of atrial remodelling caused by atrial cardiomyopathy,^4^ and impaired left atrial function, as assessed by cardiac magnetic resonance imaging, predicts the prevalence of LVAs.^18^ Low-voltage areas are also commonly arrhythmogenic in AF patients,^19,20^ and conversely, AF burden is associated with both progressive and regressive changes in LVAs.^6,21,22^ In this study, we found that AF recurrence was reduced by LVA ablation after PVI in patients with CKD G3a-5.

We suggest several possible explanations for this finding. First, the arrhythmogenicity of LVA may be particularly high in patients with CKD, given that arrhythmogenicity in LVA lesions may also be exacerbated by factors specific to renal dysfunction. For instance, renal dysfunction typically involves electrolyte and pH abnormalities such as hyperkalaemia and metabolic acidosis, as well as the accumulation of uremic toxins such as indoxyl sulfate and fibroblast growth factor-23, all of which promote arrhythmogenicity.^23^ Ablation of LVAs with high arrhythmogenicity may have an impact on reducing AF recurrence after catheter ablation.

Second, patients with renal dysfunction often demonstrate extensive LVAs, which reflect advanced atrial remodelling and are associated with an increased risk of AF recurrence following catheter ablation.^24^ Aggressive substrate modification by LVA ablation in these cases may therefore be effective. The severe atrial remodelling in patients with renal dysfunction is attributable not only to the renal dysfunction itself but also to the conditions underlying the renal dysfunction, such as hypertension and diabetes mellitus, which are also causes of atrial remodelling.^25^ Indeed, our patients with CKD G3a-5 had a greater LVA size than those with CKD G1-2, and LVA size increased with increasing CKD severity. In addition, in patients with CKD G3a-5, a greater reduction in the primary endpoint was seen in the PVI + LVA-ABL group than in the PVI-alone group in patients with large LVA size.

In contrast, in patients with CKD G1-2, rhythm outcomes after catheter ablation did not differ between the PVI + LVA-ABL group and PVI-alone group. Proarrhythmic lesions and iatrogenic atrial tachycardia may occur as side effects of additional left atrial ablation other than PVI.^1^ The arrhythmogenicity of LVA in patients with normal renal function may not reach a level at which the efficacy of substrate modification is outweighed by its side effects.

In the subgroup analysis of primary outcome in patients with CKD G3a-5, the efficacy of LVA ablation after PVI in reducing AF recurrence was decreased in patients with heart failure symptoms ≥ NYHA class II and in those with left atrial diameter ≥ 45 mm. Patients with heart failure symptoms and those with large left atrial diameter have structural atrial remodelling, including mainly left atrial enlargement.^26,27^ Low-voltage area ablation, which targets electrical atrial remodelling, might therefore not have sufficient efficacy to improve rhythm outcomes.

### Clinical implications of this sub-analysis

Over the past few decades, various ablation strategies beyond PVI have been investigated to improve rhythm outcomes following catheter ablation for persistent AF.^28^ Initially, linear ablation and/or complex fractionated electrogram ablation were commonly employed.^3,28,29^ However, LVA ablation has recently garnered attention because the STAR AF II trial demonstrated no significant improvement in rhythm outcomes with linear ablation and/or complex fractionated electrogram ablation.^3,28^ Although the primary findings of the SUPPRESS-AF trial did not reveal the efficacy of routine LVA ablation after PVI,^9^ this sub-analysis showed that AF recurrence after catheter ablation was reduced by LVA ablation after PVI in patients with renal dysfunction. These results suggest that aggressive substrate modification by LVA ablation may be considered. However, when pulsed-field ablation is employed, both renal dysfunction and a large number of applications have been identified as risk factors for post-procedural increases in serum creatinine.^30,31,32^ Therefore, the number of applications should be carefully managed. In addition, we expect that future studies will investigate whether the prevention of further CKD-induced atrial remodelling using mineralocorticoid receptor antagonists and sodium-glucose cotransporter 2 inhibitors may also improve rhythm outcomes after catheter ablation.^23,33^

Regarding patients with CKD G1-2, freedom from AF recurrence was similar between the PVI-alone and PVI + LVA-ABL groups regardless of multiple ablation procedures. Further, no subgroup in the PVI + LVA-ABL group experienced a reduction in AF recurrence compared with the respective subgroup in the PVI-alone group. Moreover, procedure time was longer in the PVI + LVA-ABL group than in the PVI-alone group, and in the main paper of the SUPPRESS-AF trial, peri-procedural adverse events tended to occur more frequently in the PVI + LVA-ABL group than in the PVI-alone group.^9^ Routine LVA ablation after PVI may not be recommended in patients with normal renal function; rather, alternative strategies for patients with LVAs and normal renal function, such as strict management of pathophysiological risk factors (e.g. hypertension, diabetes mellitus, obesity, alcohol excess, and heart failure) to prevent further atrial remodelling, may warrant exploration.^34,35^

### Limitations

This sub-analysis of the SUPPRESS-AF trial has several limitations. First, we did not have data on the underlying disease of renal dysfunction, such as diabetic nephropathy and nephrosclerosis. Additionally, we assessed eGFR only immediately before ablation and did not evaluate changes in renal function during follow-up. Second, patients on dialysis were excluded from the SUPPRESS-AF trial, so we may not have been able to accurately assess the efficacy and the safety of LVA ablation in patients with end-stage renal failure.^36^ Third, because AF recurrence was not evaluated by continuous rhythm monitoring, such as by implantable cardiac monitoring or a cardiac implantable electronic device, some AF recurrence might have been missed. Fourth, 22% of patients did not undergo complete LVA ablation, so the effects of LVA ablation might not be the same in each patient. Finally, the statistical analysis in the present study might have been weakened by the small number of patients with CKD G3b-5, adverse events, and procedure-related complications. For example, in CKD G3b-5 patients, although the rate of freedom from the primary endpoint was 25.8% higher in the PVI + LVA-ABL group than in the PVI-alone group, this difference was not statistically significant.

## Conclusions

In patients with renal dysfunction, LVA ablation after PVI reduced AF recurrence following catheter ablation in patients undergoing radiofrequency catheter ablation for persistent AF.

## Supplementary Material

euaf205_Supplementary_Data

## Data Availability

The data underlying this article cannot be shared publicly due to for the privacy of individuals that participated in the study. The data will be shared on reasonable request to the corresponding author.

## Appendix

The OCVC-Arrhythmia Investigators: Yasuhiro Matsuda, Masaharu Masuda, and Toshiaki Mano, Kansai Rosai Hospital, Amagasaki, Japan; Masato Okada, Nobuaki Tanaka, Koji Tanaka, and Yuko Hirao, Sakurabashi Watanabe Advanced Healthcare Hospital, Osaka, Japan; Masato Kawasaki, Tetsuya Watanabe, and Takahisa Yamada, Osaka General Medical Center, Osaka, Japan; Takashi Kanda, Hitoshi Minamiguchi, Nobuhiko Makino, and Yoshiharu Higuchi, Osaka Police Hospital, Osaka, Japan; Yasuharu Matsunaga, Yasuyuki Egami, Masami Nishino, and Jun Tanouchi, Osaka Rosai Hospital, Sakai, Japan; Taiki Sato, Hirota Kida, Akihiro Sunaga, Tomoaki Nakano, Kentaro Ozu, Yohei Sotomi, Tomoharu Dohi, Katsuki Okada, Takafumi Oka, Toshihiro Takeda, Daisaku Nakatani, Shungo Hikoso, and Yasushi Sakata, Osaka University Graduate School of Medicine, Suita, Japan; Miwa Miyoshi, Osaka Hospital, Japan Community Healthcare Organization Osaka, Japan; Koichi Inoue and Yasushi Matsumura, National Hospital Organization Osaka National Hospital, Osaka, Japan; and Tomoko Minamisaka and Shiro Hoshida, Yao Municipal Hospital, Yao, Japan.
