# Supplementary material for: Impact of renal function on the efficacy of low-voltage area ablation after pulmonary vein isolation: a sub-analysis of the SUPPRESS-AF trial
Source: Europace. 2025 Sep 2;27(9):euaf205. doi: 10.1093/europace/euaf205 (PMC12448949; doi:10.1093/europace/euaf205)
Supplement: euaf205_Supplementary_Data [file euaf205_supplementary_data.zip › CKD_supplementary table_SAF subanalysis.docx]

**Supplementary table 1. Adverse events**

| **Variable** | **All**  **(n = 341)** | **CKD G1-2 (n = 172)** | | **p** | **CKD G3a-5 (n = 169)** | | **p** |
| --- | --- | --- | --- | --- | --- | --- | --- |
|  |  | **PVI-alone**  **(n = 84)** | **PVI+LVA-ABL**  **(n = 88)** |  | **PVI-alone**  **(n = 87)** | **PVI+LVA-ABL**  **(n = 82)** |  |
| Composite endpoint,^†^ n (%) | 16 (5) | 3 (4) | 7 (8) | 0.33 | 3 (3) | 3 (4) | 1.00 |
| All-cause death, n (%) | 4 (1) | 1 (1) | 2 (2) | 1.00 | 1 (1) | 0 (0) | 1.00 |
| Symptomatic stroke, n (%) | 7 (2) | 2 (2) | 4 (5) | 0.68 | 1 (1) | 0 (0) | 1.00 |
| Bleeding, n (%) | 8 (2) | 1 (1) | 3 (3) | 0.62 | 1 (1) | 3 (4) | 0.36 |

CKD = chronic kidney disease, PVI = pulmonary vein isolation, LVA = low voltage area,

^†^ Composite endpoint consisted of all-cause death, symptomatic stroke and bleeding
