# Supplementary material for: Impact of renal function on the efficacy of low-voltage area ablation after pulmonary vein isolation: a sub-analysis of the SUPPRESS-AF trial
Source: Europace. 2025 Sep 2;27(9):euaf205. doi: 10.1093/europace/euaf205 (PMC12448949; doi:10.1093/europace/euaf205)
Supplement: euaf205_Supplementary_Data [file euaf205_supplementary_data.zip › CKD_figure S 1_SAF subanalysis.pdf]

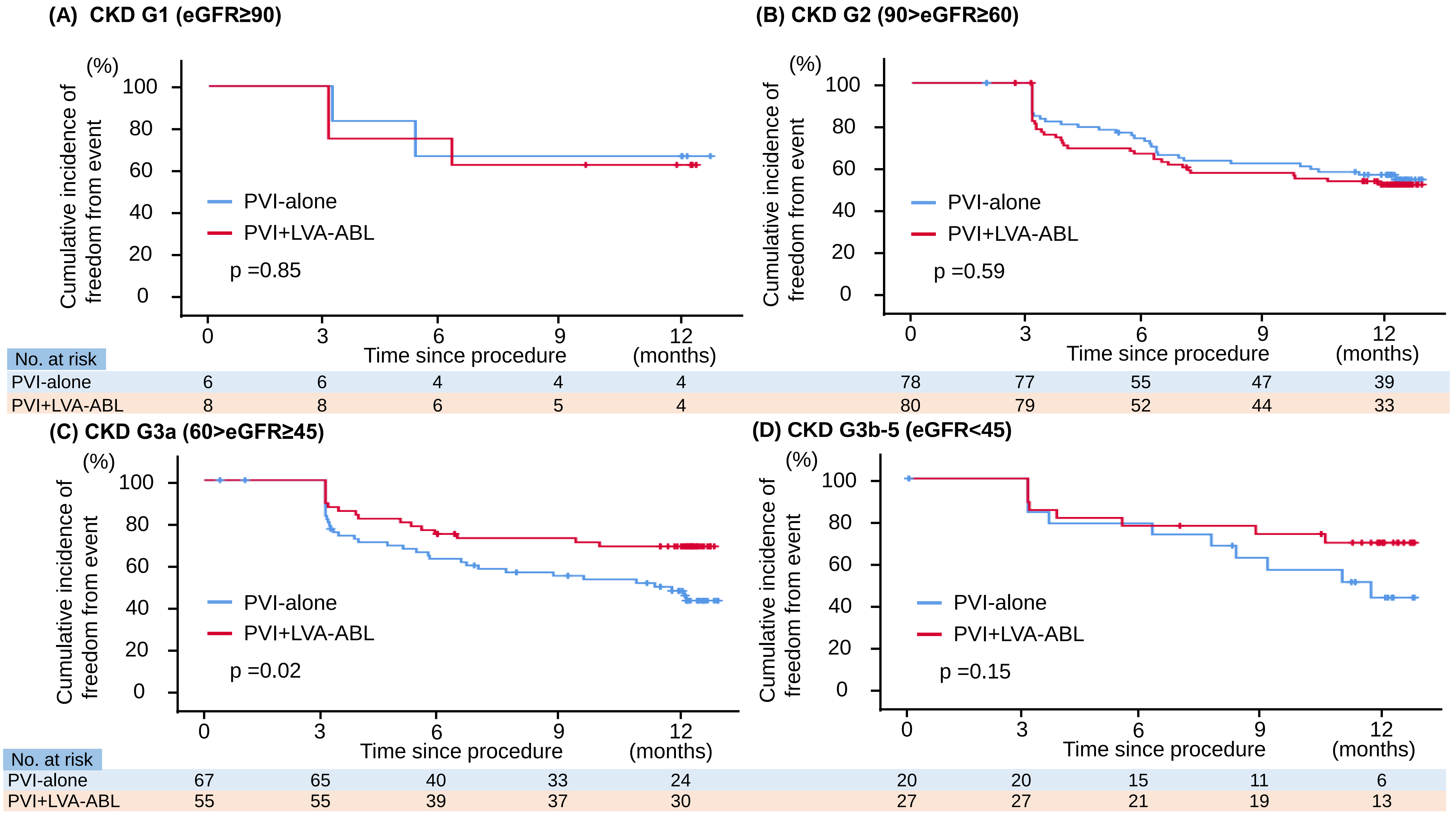
(A) CKD G1 (eGFR≥90)
(%)
100
80
60
40
20
0
Cumulative incidence of freedom from event
PVI-alone
PVI+LVA-ABL
p =0.85
0
3
6
9
12
Time since procedure
(months)
No. at risk
PVI-alone 6 6 4 4 4
PVI+LVA-ABL 8 8 6 5 4
(B) CKD G2 (90>eGFR≥60)
(%)
100
80
60
40
20
0
Cumulative incidence of freedom from event
PVI-alone
PVI+LVA-ABL
p =0.59
0
3
6
9
12
Time since procedure
(months)
78 77 55 47 39
80 79 52 44 33
(C) CKD G3a (60>eGFR≥45)
(%)
100
80
60
40
20
0
Cumulative incidence of freedom from event
PVI-alone
PVI+LVA-ABL
p =0.02
0
3
6
9
12
Time since procedure
(months)
No. at risk
PVI-alone 67 65 40 33 24
PVI+LVA-ABL 55 55 39 37 30
(D) CKD G3b-5 (eGFR<45)
(%)
100
80
60
40
20
0
Cumulative incidence of freedom from event
PVI-alone
PVI+LVA-ABL
p =0.15
0
3
6
9
12
Time since procedure
(months)
20 20 15 11 6
27 27 21 19 13
