# Supplementary figures and images for: Impact of renal function on the efficacy of low-voltage area ablation after pulmonary vein isolation: a sub-analysis of the SUPPRESS-AF trial
Source: Europace. 2025 Sep 2;27(9):euaf205. doi: 10.1093/europace/euaf205 (PMC12448949; doi:10.1093/europace/euaf205)

(A) CKD G1-2 (eGFR≥60)

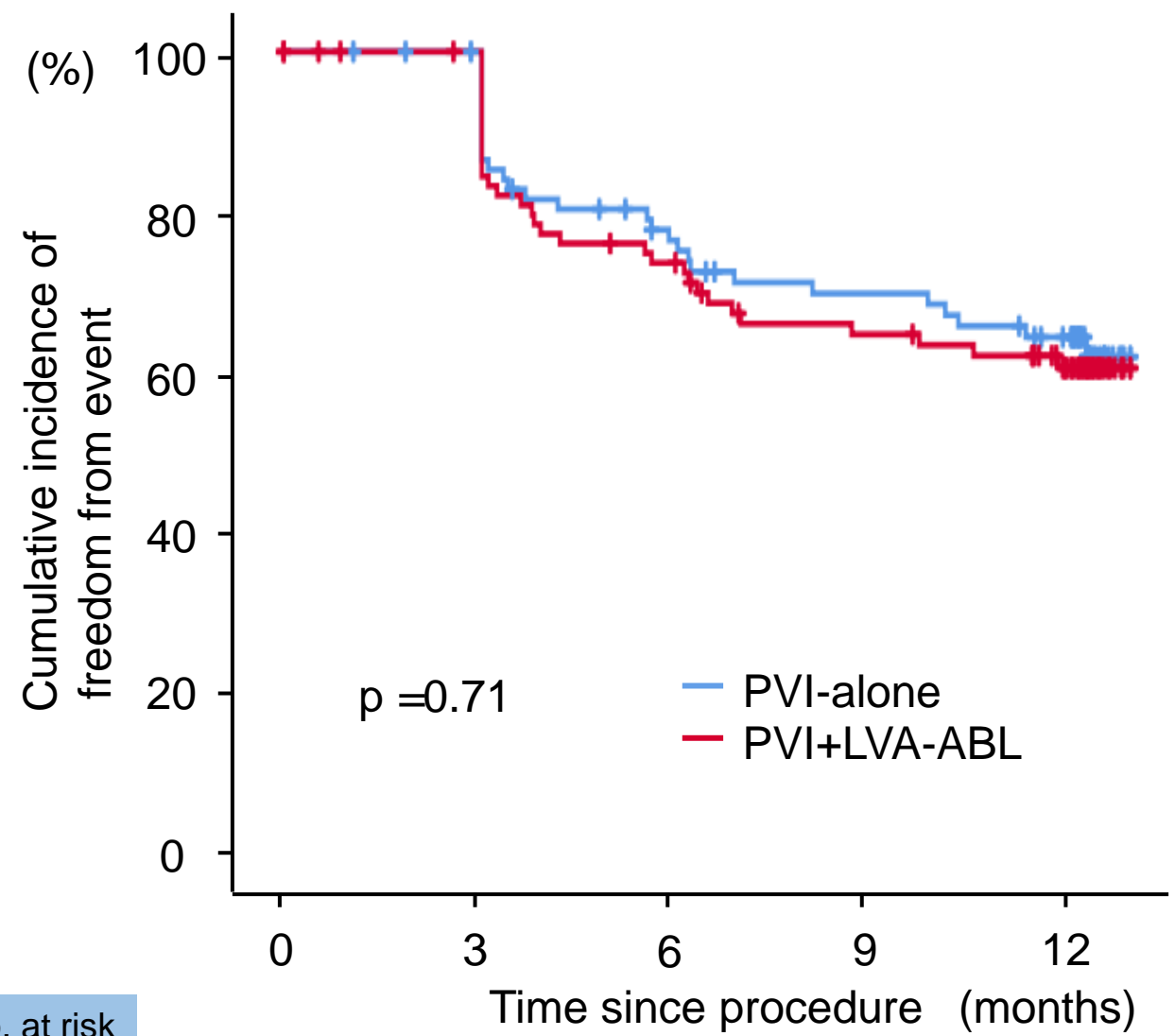

(B) CKD G3a-5 (eGFR<60)

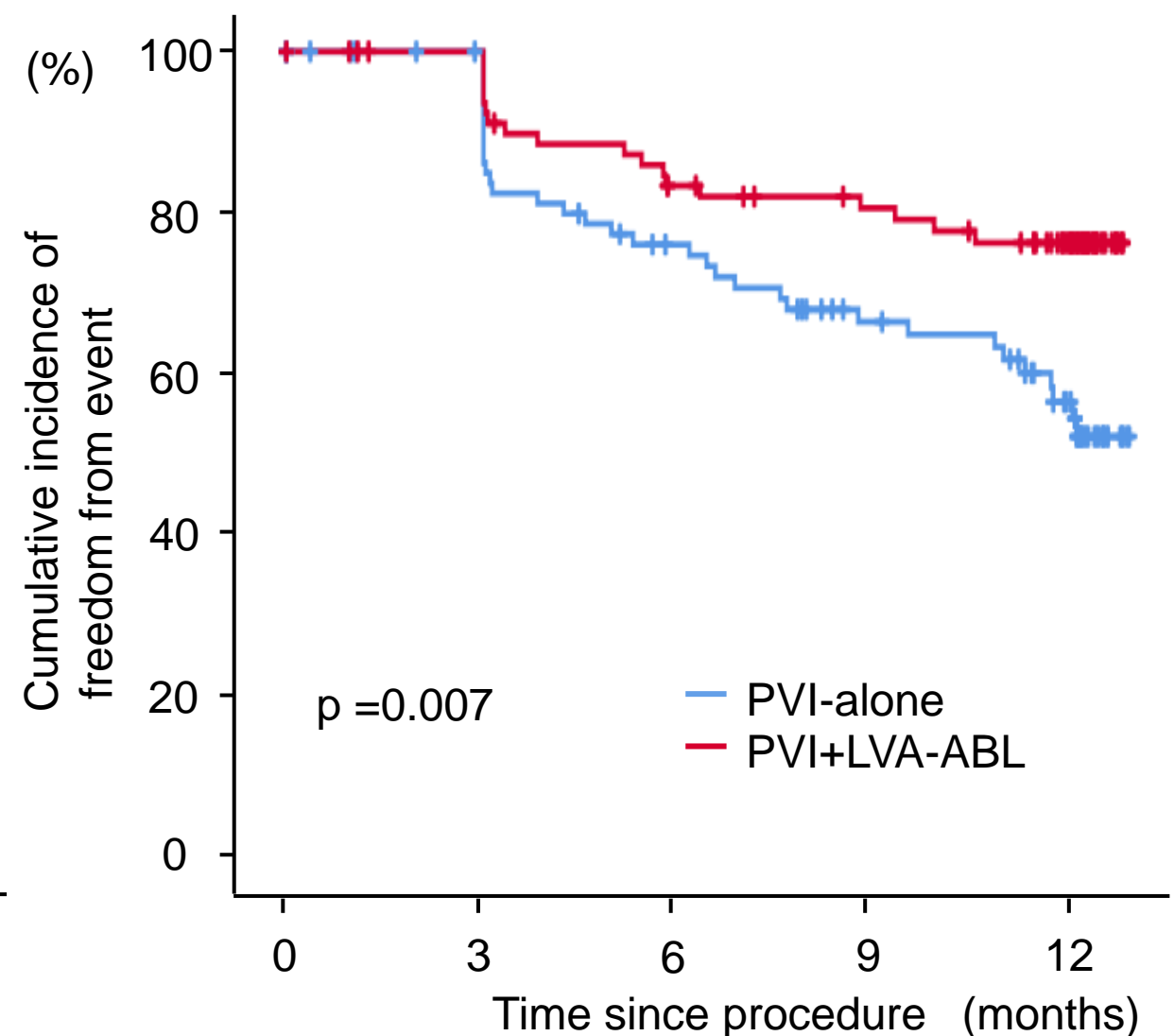

| No. at risk | 0  | 3  | 6  | 9  | 12 | 0  | 3  | 6  | 9  | 12 |
|-------------|----|----|----|----|----|----|----|----|----|----|
| PVI-alone   | 84 | 81 | 58 | 51 | 43 | 87 | 79 | 56 | 43 | 30 |
| PVI+LVA-ABL | 88 | 83 | 60 | 49 | 38 | 82 | 78 | 62 | 56 | 43 |

Supplement: euaf205_Supplementary_Data [file euaf205_supplementary_data.zip › CKD_figure S 2_SAF subanalysis.pdf]
